# Personality Traits and Career Role Enactment: Career Role Preferences as a Mediator

**DOI:** 10.3389/fpsyg.2019.01720

**Published:** 2019-07-25

**Authors:** Nicole de Jong, Barbara Wisse, José A. M. Heesink, Karen I. van der Zee

**Affiliations:** ^1^Department of Psychology, University of Groningen, Groningen, Netherlands; ^2^Durham University Business School, Durham, United Kingdom; ^3^Faculty of Social Sciences, Vrije Universiteit Amsterdam, Amsterdam, Netherlands

**Keywords:** personality, career role preferences, career roles, career role enactment, job crafting

## Abstract

It has been argued that how a person’s career unfolds is increasingly affected by his or her own values, personality characteristics, goals and preferences. The current study addresses the issue of how we can explain that personality traits are associated with the enactment of certain career roles. Two survey studies (e.g., a two wave worker sample and a cross-sectional worker sample) were conducted to investigate the relationships between personality traits, career role preferences and career role enactment. As expected, results indicate that peoples’ personality traits predicted the preference for certain roles in the work context which, in turn, predicted the career roles they actually occupy. Specifically, our findings show that Extraversion, Conscientiousness and Openness to experience influence various career role preferences (i.e., Maker, Expert, Presenter, Guide, Director, and Inspirer role preferences) and, subsequently, the enactment of these career roles. Other traits, such as Neuroticism and Agreeableness, seem less important in predicting role preferences and subsequent role enactment. These results underline the importance of acknowledging not only individual trait differences but especially role preferences in explaining how careers develop over time. Further implications, limitations and research ideas are discussed.

## Introduction

Nowadays, employees often can autonomously change, adapt, modify and tailor their jobs or the way in which they perform their jobs (Parker, 2000; Organ et al., 2006; Oldham and Hackman, 2010). The question as to what determines how individuals customize their jobs, and ultimately their careers, has received increasing research attention (Parker and Bindl, 2017). Several scholars have argued that how a person’s career unfolds is strongly affected by his or her own values, personality characteristics, goals and preferences (Hall, 2004; Wille et al., 2012; Savickas, 2013). The Big Five trait taxonomy (McCrae and Costa, 1996, 1999; also see the Five Factor Model [FFM], Goldberg, 1990) appears to offer a particularly promising approach to the application of personality constructs to career related outcomes. Indeed, the Big Five is an empirically validated classification of the structure and nature of personality traits. Its usefulness for predicting job crafting behavior and career role enactment is apparent from several studies that show personality traits do indeed affect the way in which people perform their jobs over time (Wille et al., 2010, 2012; Bakker et al., 2012).

**FIGURE 1:**
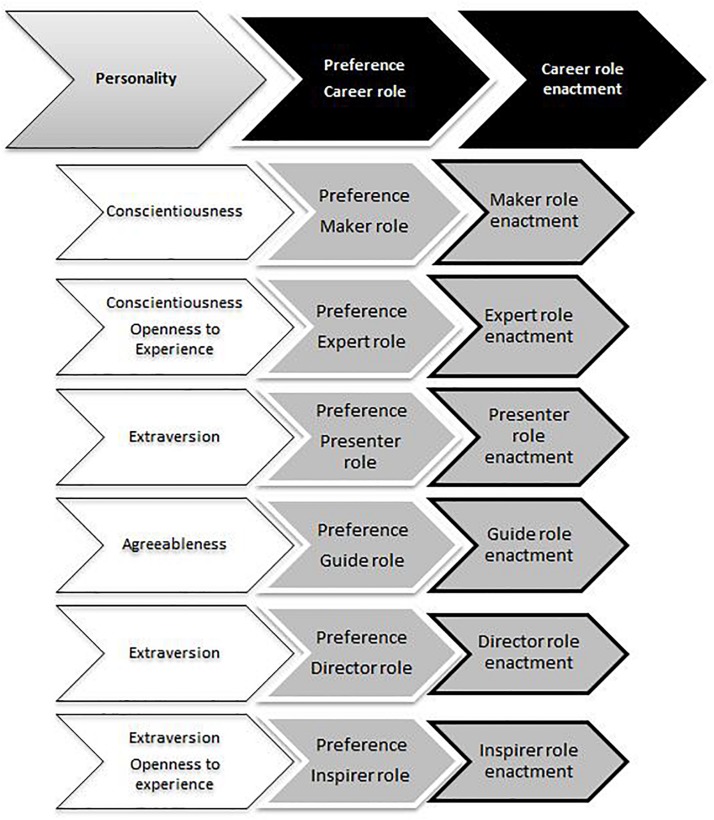
Conceptual model of how personality traits relate to career role preferences and career role enactment.

The current study also explores the relationship between personality traits and career role enactment, but includes a potential mediating mechanism. Indeed, previous research has left the question as to how we can explain that personality traits are associated with the enactment of certain career roles largely unanswered. In line with the functionalist approach to personality (Wood et al., 2015), we argue that each personality trait engenders a preference for certain career roles. These preferences, in turn, will affect individuals’ behaviors and thus also the likelihood that certain career roles will eventually be enacted. Therefore, we propose that career role preferences will function as a mediating mechanism in the relationship between the Big Five personality traits and career role enactment (see Figure 1).

With this study, we hope to contribute to the existing literature in several ways. First, this study answers the call for more research to explain the personality traits – behavior at work relationship (see Barrick, 2005). Understanding the underlying mechanisms that clarify the relationship between personality traits and career role enactment may not only contribute to the development of personality theory, but may also help us to identify factors that more directly influence career role enactment (i.e., the proposed mediators). Second, we hope to contribute to the growing body of research that acknowledges that employees are not passive recipients of job characteristics who walk fixed career paths, but instead can be seen as active agents in the construction of their work and career (Savickas, 2013; Wrzesniewski et al., 2013). By investigating how traits and career role preferences affect career role enactment, this study highlights how employees have a determining role in their own in career development. Insights into these matters can contribute to employee perceptions of control over the work environment and perceptions of self-efficacy and competence (Spreitzer and Doneson, 2005). Finally, we hope that the insights derived from this study may offer some tentative practical suggestions to employees who want to plan their career, as well as to HRM-practitioners, coaches and others who are interested in providing guidance and support to individual employees.

### Career Role Enactment and Career Role Preferences

In order to understand individual career development, the Career Roles Model was developed (Hoekstra, 2011). This model builds on the notion that nowadays jobs can’t easily be defined by a set list of specific tasks. Instead, jobs have become more complex and can often be better described by work roles (Huckvale and Ould, 1995). Work roles include tasks, but are more broad and also incorporate processes, responsibilities and functions that may change based on needs and opportunities that arise (Huckvale and Ould, 1995). The Career Roles Model states that people enact different work roles in their jobs. Over time, these work roles may grow into enduring *career roles* (Hall, 1976; Hoekstra, 2011). Career roles can be defined as stable and repetitive patterns of functioning in the work context that are independent of specific jobs and levels of functioning. Carrying out a role has been referred to as role enactment (Hoekstra, 2011; De Jong et al., 2014). *Career role enactment* can thus be seen as the behavioral manifestation of occupying certain career roles (i.e., the actual engaging in these roles).

The Career Roles Model (see Supplementary Table A1) identifies six different career roles. These roles are based on the systematic combination of three classes of individual motives that drive people in their work and two organizational themes that guide organizations. The classes of individual motives, derived from Hogan (2007), are *distinction* (e.g., autonomy and agency), *integration* (e.g., connectedness and belonging) and *structure* (e.g., collective meaning and cohesion). These classes are crossed with two organizational themes: *exploitation* (e.g., processes focused on stability) and *exploration* (e.g., processes aimed at innovation and change) derived from March (1999). The six resulting roles are (1) the Maker role; (2) the Expert role; (3) the Presenter role; (4) the Guide role; (5) the Director role; and (6) the Inspirer role. According to the Career Roles Model, these six roles are the building blocks of individual careers and potentially attainable in most jobs with at least some employee autonomy (Hoekstra, 2011).

*Career role preferences* are defined as “the mental act of identifying with the career role as part of the self” (De Jong et al., 2014. p. 201). A career role that is preferred is seen as more fitting to the self and more attractive and desirable than a career role that is not preferred. Thus, whereas a career role preference concerns the extent to which individuals like to see themselves in a certain light, career role enactment concerns the performing of acts that are associated with that role. We posit that career role preferences will serve as a mediating mechanism in the personality traits – career role enactment relationship. Before we turn to more specific hypotheses, we will elaborate on insights from personality theory that substantiate this general proposition.

### Personality Traits and Their Relationship With Preferences and Behavior

Personality traits are aspects of personality that are relatively stable over time, differ across individuals and are relatively consistent over situations (Anusic and Schimmack, 2016). Probably the most common framework to the study of personality traits is the Big Five. The Big Five trait taxonomy is a hierarchical model of personality traits with five broad factors, which represent personality at the broadest level of abstraction. These factors are Neuroticism, Extraversion, Openness to Experience, Agreeableness, and Conscientiousness. Each factor describes a broad domain of psychological functioning that is composed from a set of more specific and narrow traits (Roberts et al., 2006). Several scholars have argued that personality traits, processes and behaviors should be separated from each other in order to increase our understanding of how personality explains behaviors (see Baumert et al., 2017; Zeigler-Hill et al., 2019). In this perspective, personality traits can be seen as basic tendencies or general predispositions, largely controlled by biological influences (McCrae and Costa, 2008; McCrae, 2018). In contrast, motivational processes, such as preferences, as well as subsequent behaviors, represent the interaction between personality traits and the specifics of the social context. As such, processes and behaviors give contextualized form to what it means to possess certain relatively broad and abstract personality traits (e.g., Cantor, 1990; McAdams and Pals, 2006; McCrae and Costa, 2008; Wood et al., 2015; McCrae, 2018). Preferences, therefore, can be seen as being a consequence of inherent personality traits. These preferences will, in turn, affect a person’s behavior. Specifically, an individual’s behavioral displays are expected to follow the *law of effect*: a certain behavior increases when it satisfies a person’s needs and desires, and a certain behavior decreases when it does not (see Wood et al., 2015). That is, a certain behavior will be displayed more often if that behavior is congruent with a person’s preferences (c.f., Trait activation theory; Christiansen and Tett, 2008). All in all, one could argue that personality traits affect a person’s preferences, and that these preferences will guide that person’s behavior in such a way that it will be beneficial and satisfying to the person.

Notably, several studies support the perspective that motivational processes (e.g., preferences, goals, motives) mediate the associations between Big Five personality traits and behavior relevant to organizational functioning, such as counterproductive work behavior (Mount et al., 2006), job performance (Barrick et al., 2002), creative achievement (Prahbu et al., 2008), volunteering (Carlo et al., 2005), career decision making (Shafer, 2000), and training performance (Major et al., 2006).

### The Influence of Personality Traits in the Context of Career Roles

If we translate the above theoretical insights to our current research, it suggests that, depending on a person’s traits, some career roles will seem more attractive and desirable than others. If a certain career role is preferred, people will start to behave in a way that will allow them to engage in that role. Engaging in the role is likely to be intrinsically satisfying, because people are likely to feel good about being able to express their traits in their work environments. Arguably, certain roles allow people to express their traits more than others. Therefore, although restrictions by external demands or expectations can always occur, in general people’s preferences will influence their role-taking behavior or their role enactment (Parker et al., 2010; De Jong et al., 2014).

So far, one of the few studies to investigate the link between personality traits and career roles was conducted by Wille et al. (2012). Wille et al. (2012) specifically investigated the relationship between the Big Five personality traits and career role enactment. A sample of college alumni provided self-reports on their Big Five personality traits 3 months prior to graduation and 15 years later when their career had unfolded. Results indicated significant positive associations between several personality traits and (changes in) career role engagement. Individuals scoring high on Conscientiousness reported stronger engagement in the Expert role, Extraverts scored higher on the Presenter, Guide, Director and Inspirer roles. Agreeableness was predictive of stronger Guide role engagement and Openness was (unexpectedly) positively related to Presenter role engagement. Interestingly, Neuroticism did not relate significantly to engagement in any of the career roles. This study provides an excellent starting point for additional research on the matter. For instance, in the Wille et al. (2012) study, respondents were asked to retrospectively report on the importance of certain career roles over time. Confirmation of results from studies using different designs would bolster confidence in the findings. Moreover, in the Wille et al. (2012) study, potential mediating mechanisms between personality and career role enactment were not investigated, although they argue that such endeavors would be welcome (see p. 319). The current study addresses both issues by performing two studies with a different design (one two-wave and one cross-sectional design) and by examining the potential mediating role of career role preferences in the relationship between personality characteristics and career roles. In sum, the current study focuses on how specific personality characteristics relate to career role preferences and subsequently result in career role enactment (see Figure 1). In the following, we will discuss each of the career roles outlined in the Career Roles Model (Hoekstra, 2011) and how these are expected to be related to individual preferences and the Big Five personality traits.

### Hypotheses

First, the Maker role can be characterized by the striving for personal goals such as individual mastery and success. The role has a strong emphasis on autonomy and independence (Hoekstra, 2011). People that occupy the Maker role do well in an environment with clear guidelines and task descriptions. We argue that those who score high on Conscientiousness may be more likely to end up in the Maker role, because this personality trait engenders in people a preference for tasks in which they may demonstrate the will to achieve (Digman and Inouye, 1986), to work hard, and to be responsible well organized (Wille et al., 2013). Indeed, Conscientiousness is expected to be related to a preference for tasks in which people can show that they are agentic and responsible (Chiaburu et al., 2011), which in turn will foster enactment of the Maker role.

The Expert role is also characterized by personal goal setting, with a strong emphasis on independent agency. Additionally, the Expert role is considered to be accompanied by a natural eagerness to explore. People in the Expert role therefore typically engage in problem solving behavior (Hoekstra, 2011). Because Conscientiousness stimulates in people a preference for tasks in which they may demonstrate the will to achieve, we expect that Conscientiousness will also related to a preference for and subsequent enactment of the Expert role (Wille et al., 2012). Additionally, both people who score high on Conscientiousness, as well as people who score high on Openness to experience, score relatively high on problem solving ability (D’Zurilla et al., 2011) which may boost their preference for such role. Openness to experience is also often associated with the ability to think outside the box and with being curious and unconventional (Barrick et al., 2003; Fuller and Marler, 2009), as well with a growth tendency and the ability to adapt (Digman, 1997; Lepine et al., 2000), which may stimulate their preference for the Expert role, and therefore their subsequent of the Expert role.

*Hypothesis 1* The positive relationship between Conscientiousness and career role enactment of the Maker role is mediated by preference for the Maker role.
*Hypothesis 2a* The positive relationship between Conscientiousness and career role enactment of the Expert role is mediated by preference for the Expert role.
*Hypothesis 2b* The positive relationship between Openness to experience and career role enactment of the Expert role is mediated by preference for the Expert role.

The Presenter role can be characterized by the focus on social interactions. Typically, people in this role engage in activities in which they influence others, for example as a sales person or a lawyer (Hoekstra, 2011). In line with Wille et al. (2012), we expect people who score high on Extraversion to be attracted to the Presenter role, because people scoring high on Extraversion are often dominant, active and assertive (Wiggins and Broughton, 1985; Barrick et al., 2003) which matches nicely with the social influence aspect of the Presenter role. Thus, Extraversion is expected to be related to a preference for roles in which one may persuade and influence others (Oh and Berry, 2009), which in turn is likely to be positively related to enactment of the Presenter role.

Characteristic for the Guide role is that behaviors take place in social settings and revolve around social interactions (e.g., connecting and cooperating with colleagues). However, in the Guide role the focus is not so much on influence and persuasion (as it is in the Presenter role), but on helping and guiding others while maintaining focus on others’ perspectives (Hoekstra, 2011). Based on these characteristics of the Guide role, and in line with Wille et al. (2012), we believe that people scoring high on Agreeableness/Friendliness will end up in the Guide role. Agreeableness/Friendliness is characterized by a tendency to be warm, kind and unselfish (Costa and McCrae, 1997; Barrick et al., 2003). Furthermore, agreeable individuals will have a preference for harmonious interpersonal environments (Barrick et al., 2002). Therefore, people who score high on Agreeableness/Friendliness will show a preference for seeing oneself as someone who is directed toward helping others and being cooperative, which in turn will enhance Guide role enactment.

*Hypothesis 3* The positive relationship between Extraversion and career role enactment of the Presenter role is mediated by preference for the Presenter role.
*Hypothesis 4* The positive relationship between Agreeableness/friendliness and career role enactment of the Guide role is mediated by preference for the Guide role.

The Director role is typified by activities focused on optimizing strategy and by minding the overarching structure of groups and organizations (Hoekstra, 2011). In line with Wille et al. (2012), we believe that especially those people who score high on Extraversion will prefer the Director role, as they have the opportunity to realize, establish and choose long-term goals from a dominant position (Paulhus and John, 1998; Hogan and Holland, 2003). Indeed, as previous research has demonstrated, Extraversion is positively associated with a sensitivity to potential rewards (Lucas et al., 2000) and dominance (Barrick et al., 2003). Therefore, it is expected that Extraversion is positively associated with a preference for the Director role, which in turn will result in Director role enactment.

Finally, similar to the Director role, the Inspirer role is also characterized by the tendency to focus on optimizing strategy. However, people in the Inspirer role are predominantly concerned with initiating strategic change away from current strategic programs, (often) without formal authority (Hoekstra, 2011). We believe that people who score high on Openness to experience are more likely to a preference for tasks that involve non-conformity and abstraction (Barrick et al., 2003). Moreover, Openness to experience comes with the ability to be unconventional and think outside the box (Costa and McCrae, 1992), both of which are important for initiating change in new directions. Furthermore, especially when lacking formal authority, inspiring others to embrace change initiatives is more likely when it is done in an energetic, assertive fashion and with the display of positive emotions (Bono and Judge, 2004). As mentioned, extraverted people are relatively dominant, active and assertive (Wiggins and Broughton, 1985; Barrick et al., 2003). Therefore, we believe that both Openness to experience and Extraversion will result in a preference for the Inspirer role, which in turn will foster enactment of the Inspirer role.

*Hypothesis 5* The positive relationship between Extraversion and career role enactment of the Director role is mediated by preference for the Director role.
*Hypothesis 6a* The positive relationship between Extraversion and career role enactment of the Inspirer role is mediated by preference for the Inspirer role.
*Hypothesis 6b* The positive relationship between Openness to experience and career role enactment of the Inspirer role is mediated by preference for the Inspirer role.

### Overview of the Studies

To test our hypotheses, we conducted two studies. Study 1 is a two-wave survey of US workers. In Wave 1, we assessed employees’ Big Five personality traits using the Big Five Inventory (BFI, John et al., 1991, see also Benet-Martinez and John, 1998; John and Srivastava, 1999). In Wave 2, we assessed employees’ career role preferences using vignettes that were based on the CRIQ 1.0 (De Jong et al., 2014) and career role enactment using the VLR-30 (Hoekstra, 2011). Study 2 is a cross-sectional survey of Dutch workers. In this study, we assessed employees’ Big Five personality traits using the G5short (Hiemstra et al., 2011), their career role preferences with the CRIQ 1.0, and their career role enactment with the VLR-30. The advantage of the two-wave study is that it may be less subjected to problems related to multicollinearity. Moreover, the studies sampled from different populations and used instruments to assess personality traits that fitted that specific population (developed for people from English and Dutch speaking populations, respectively). To measure career role preferences, we used in both studies the CRIQ 1.0. However, whereas in Study 1 we grouped items and wrote them into vignettes (arguably making it easier for respondents to differentiate among the various preferences), we used separate items in Study 2. All in all, by replicating our findings over studies with different research methods and samples, we aimed to bolster the confidence in our results (Shadish et al., 2002).

## Method Study 1

### Respondents and Procedure

A two-wave online survey study with employees from the United States was conducted. In total, 279 employees^1^ completed both waves (*M*_age_ = 39.11, *SD*_age_ = 10.80, 49% female). Employees working a minimum of 24 (payed) hours a week were allowed to participate in the survey. Of the employees, 1.1% completed primary school, 18.3% completed secondary school, 18.3% completed technical secondary school, 48% completed a bachelor’s program, and 14.3% completed a master’s program or higher. Furthermore, for their current job 14.3% of the participants required little to no training, 19.7% required a few months to a year training, 28% required 1–2 years training, 31.2% required a considerable amount of training including several years of work-related experience, and 6.8% required extensive skill, knowledge and more than 5 years of experience. Average employment in the labor market was 18.38 years (*SD* = 11.23).

After having received study approval from the ethics committee of the University, we recruited employees via the online platform Amazon Mechanical Turk. Previous studies have shown that Mechanical Turk data are as reliable as traditional survey samples, specifically when measures to increase data quality are taken into account (Cheung et al., 2017; Keith et al., 2017; Buhrmester et al., 2018). Participants were briefed about the content of the study, the voluntary nature of the study and confidentiality before giving their informed consent. In Wave 1, employees completed a questionnaire that assessed demographic variables and personality traits. After 3 weeks, in Wave 2, career role preferences and career role enactment data were collected. Participation in each of the waves took approximately 15 min and employees received 1.75 US$ upon completion of both studies.

### Materials

#### Personality Traits

Personality traits were measured using the Big Five Inventory (BFI, John and Srivastava, 1999). The BFI contains 44-items and assesses Neuroticism, Extraversion, Conscientiousness, Agreeableness, and Openness to experience^2^. Respondents were asked to indicate to what extent they agreed (1 = *strongly disagree*, 5 = *strongly agree*) to statements as: *“I see myself as someone who is depressed, blue”* (Neuroticism, eight items, α = 0.92), “*I see myself as someone who is talkative”* (Extraversion, eight items, α = 0.91), “*I see myself as someone who does a thorough job”* (Conscientiousness, nine items, α = 0.89), “*I see myself as someone who is helpful and unselfish with others”* (Agreeableness, nine items, α = 0.87) and “*I see myself as someone who is original, comes up with new ideas”* (Openness to experience, ten items, α = 0.81).

#### Career Role Preferences

To assess the extent to which employees had a preference for certain career roles, we first made vignettes describing the six career roles and we presented those, in random order, to the respondents. The different vignettes were based on the items of the CRIQ 1.0 (De Jong et al., 2014). An example of a vignette is “*You want to realize your goals and you want to get concrete results. You work hard and thorough on assignments and you like to get the process going. You are often the one who takes care of the concrete realization of a project. You take action when there is work to do. In addition, you want to organize things yourself to achieve good results. You focus on routine tasks and you can perform independently of others*.” (Maker role)^3^.

Subsequently, for all vignettes, career role preferences were measured using an adaptation of the 7-item Self-brand connections questionnaire (with α’s ranging from α = 0.95 to α = 0.98, Escalas and Bettman, 2003). Employees were asked to indicate their agreement (1 = *strongly disagree*, 7 = *strongly agree*) with statements like “*This role reflects who I am,”* “*I feel a personal connection to this role,”* and *“I can identify with this role.”*

#### Career Role Enactment

Career role enactment was measured with the VLR-30 (Hoekstra, 2011). One advantage of the VLR-30 is that people’s enactment of multiple roles can be assessed. Each item of the 30-item questionnaire (five items per career role) gives an example of behavior that would fit specifically with one career role. Respondents were asked to indicate how well the item described what they would typically do at work (1 = *not at all*, 7 = *very well*) during the last year. Examples of items are “I …” “…*organize many things personally to get good results*” (Maker role, α = 0.66), “…*analyze a problem that others find complicated”* (Expert role, α = 0.82), “…*frame a plan carefully to get broad acceptance*” (Presenter role, α = 0.86), “…*gain someone’s confidence in a difficult relationship*” (Guide role, α = 0.87), “…*take the lead in confusing situations”* (Director role, α = 0.89) and “…*stimulate others’ minds with creative ideas”* (Inspirer role, α = 0.81).

#### Control Variables

Demographic variables (age, sex [0 = *men*; 1 = *women*], education [1 = *preliminary school*, 2 = *high school*, 3 = *intermediate vocational education*, 4 = *higher vocational education*, 5 = *university degree*]) were included as control variables in the analyses. Moreover, we also added years of employment in the labor market and job zone [ranging from 1 = *no to little preparation or education is needed* to 5 = *extensive preparation and education is needed*] as control variables (Becker, 2005) to guard against job complexity affecting the relationship between personality traits and career role enactment. To assess job zone, we used the classifications as provided by an online tool for career exploration and analyses (O^*^net, 2019; for similar use see Baughman et al., 2015).

## Results Study 1

### Preliminary Analyses

Correlations, means, and standard deviations of the study variables are presented in Supplementary Table A2. Note that the correlations indicate that personality traits are associated with career role preferences and career role enactment, as hypothesized. Furthermore, these results are in line with previous findings on personality and career roles (e.g., Wille et al., 2012).

### Mediation Analyses

To investigate the proposed mediating role of career role preferences in the relation between personality traits and career roles, the PROCESS macro for SPSS by Hayes (2013) was used (see Supplementary Tables A3–A8; first columns). In each analysis, the enactment of one of the six roles was added as the dependent variable, the preference for that role was added as a mediator variable and the five personality variables were added as predictor variables^4^. Furthermore, age, sex, education, years of employment in the labor market and job zone were included as a covariate in the mediation model. In general, our overarching model that specific personality traits relate to career role preferences, subsequently resulting in career role enactment (see Figure 1), is confirmed. Below, we describe the results for each of the hypotheses.

### Maker Role

Preference for the Maker role was, as expected, positively related to Maker role enactment (see Supplementary Table A3). In addition, we found a positive relation between Conscientiousness and preference for the Maker role, and between Conscientiousness and Maker role enactment. Furthermore, in line with Hypothesis 1, we found that the indirect effect of Conscientiousness via preference for the Maker role on perceived enactment of the Maker role was significant (Effect = 0.11, *SE* = 0.04, CI = [0.04; 0.20]). Other personality traits did not predict Maker role enactment.

### Expert Role

We found a significant positive relationship between preference for the Expert role and Expert role enactment. However, we did not find a significant positive relationship between Conscientiousness and preference for the Expert role and Conscientiousness and Expert role enactment (see Supplementary Table A4). Hypothesis 2a could therefore not be confirmed.

Second, we found a significant positive relationship between Openness to experience and preference for the Expert role, and between Openness to experience and Expert role enactment. Furthermore, in line with Hypothesis 2b, we found that the indirect effect of Openness to experience via preference for the Expert role on enactment of the Expert role was significant (Effect = 0.23, *SE* = 0.06, CI = [0.12; 0.36]).

### Presenter Role

For the Presenter role, we found a significant positive relationship between preference for this role and Presenter role enactment, between Extraversion and preference for the Presenter role, and between Extraversion and Presenter role enactment (see Supplementary Table A5). Furthermore, in line with Hypothesis 3, we found that the indirect effect of Extraversion via preference for the Presenter role on enactment of the Presenter role was significant (Effect = 0.10, *SE* = 0.04, CI = [0.02; 0.18]). Other personality traits did not predict Presenter role enactment.

### Guide Role

For the Guide role, we found a significant positive relationship between preference for this role and Guide role enactment and between Agreeableness and preference for the Guide role (see Supplementary Table A6). Furthermore, in line with Hypothesis 4, we found that the indirect effect of Agreeableness via preference for the Guide role on enactment of the Guide role was significant (Effect = 0.40, *SE* = 0.09, CI = [0.25; 0.59]).

Unexpectedly, we found that Neuroticism and Extraversion were positively related to preference for the Guide role and that the indirect effects of Neuroticism and Extraversion via preference for the Guide role on enactment of the Guide role were significant as well (Effect = 0.15, *SE* = 0.06, CI = [0.05; 0.27]; Effect = 0.12, *SE* = 0.05, CI = [0.03; 0.23], respectively).

### Director Role

We found a significant positive relationship between preference for this role and Director role enactment, between Extraversion and preference for the Director role and between Extraversion and enactment of the Director role (see Supplementary Table A7). Furthermore, in line with Hypothesis 5, we found that the indirect effect of Extraversion via preference for the Director role on perceived enactment of the Director role was significant (Effect = 0.11, *SE* = 0.05, CI = [0.01; 0.21]). Other personality traits did not predict Director role enactment.

### Inspirer Role

For the Inspirer role, we found a significant positive relationship between preference for this role and Inspirer role enactment (see Supplementary Table A8). Moreover, we found a significant positive relationship between Extraversion and preference for the Inspirer role and between Extraversion and Inspirer role enactment. Furthermore, in line with Hypothesis 6a, we found that the indirect effect via preference for the Inspirer role on enactment of the Inspirer role was significant (Effect = 0.11, *SE* = 0.04, CI = [0.05; 0.20]).

Second, we found a significant positive relationship between Openness to experience and preference for the Inspirer role, and between Openness to experience and Inspirer role enactment. Furthermore, in line with Hypothesis 6b, we found that the indirect effect via preference for the Inspirer role was significant (Effect = 0.13, *SE* = 0.06, CI = [0.03; 0.26]).

## Method Study 2

### Respondents and Procedure

The second study was part of a survey on career role development and employability of a Dutch worker sample. Respondents were a random sample of 285 employees from different organizations (46.1% female, *M*_age_ = 40.7, *SD*_age_ = 9.5). Of the employees, 0.7% completed primary school, 5.6% completed secondary school, 13.7% completed technical secondary school, 50.4% completed a higher vocational program and 29.6% completed a bachelor or master’s program. Furthermore, for their current job 3.2% of the participants required a few months to a year training, 15.8% required 1–2 years training, 76.7% required a considerable amount of training including several years of work-related experience, and 4.3% required extensive skill, knowledge and more than 5 years of experience. Employees’ average organizational tenure was 7.68 years (*SD* = 6.6).

Various companies (in different sectors) in the Netherlands were contacted after having received study approval from the ethics committee of the University. When organizations gave their permission, employees were invited via their work e-mail to participate in an online portal study. Participation was voluntary, not part of company policy, individual results were not shared with representatives of the participating organizations, and anonymity was guaranteed. The final sample consisted of employees from multiple organizations located in the Netherlands, representing a wide range of professions (e.g., technicians, nurses, doctors, policy makers). During the data collection phase, the respondents received multiple (e-mail) reminders. To provide an incentive for participation, respondents received a feedback report on their personality traits and career roles profile after completion of the study (Kühne and Kroh, 2016).

### Materials

#### Personality Traits

Personality was measured with the Dutch version of the G5short, a 60-item questionnaire that has shown reliability and validity as a measure of the Big Five personality dimensions (Hiemstra et al., 2011, max. 12 items per subscale). Respondents were asked to indicate the extent to which each statement was descriptive of them by moving the slider to the left (0 = *NO!*) or right (100 = *YES!*). The sliders show textual captions, not the accompanying score. Translated examples of items are: “*Enjoys meeting new people*” (Extraversion, 12 items, α = 0.91), “*Stays calm under all circumstances*” (Stability, 12 items, α = 0.87), “*Is open to the values of others*” (Openness to experience, six items, α = 0.73), “*Works systematically*” (Conscientiousness, 11 items, α = 0.87), “*Has trust in others*” (Agreeableness/Friendliness, 9 items, α = 0.77).

#### Career Role Preferences

Career role preferences were measured by the Career Role Identification Questionnaire (CRIQ 1.0, De Jong et al., 2014), a 40-item-set questionnaire (six scales, 20 item-words per scale). Each item-set contains three word-items from different career role scales. Thus, word-items referring to the same career role scale were never used in one item-set. For every word-item in the item-set we asked participants to rate on a 7-point scale: “*To what extent do the following words relate to you as a person*” ranging from 1 (*I do not relate to this word*) to 7 (*I strongly relate to this word*). Translated examples of word-items are “Make” (Maker role, α = 0.97), “Know” (Expert role, α = 0.96), “Show” (Presenter role, α = 0.94), “Connect” (Guide role, α = 0.94), “Control” (Director role, α = 0.96), and “Stimulate” (Inspirer role, α = 0.94). All Likert rating combinations are possible in every item-set (for example, 2-2-2, 5-3-1, or 7-5-3). We calculated the score for the preference for a role by adding up all responses from the word-items belonging to one career role scale.

#### Career Role Enactment

Similar to Study 1, career role enactment was measured with the VLR-30 (Hoekstra, 2011). Respondents indicated how well each of the 30 statements described the role they typically enacted in their work using a slider scale (1 = *not at all*, 100 = *very well*). The sliders only showed the caption, not the accompanying score. We calculated the mean score for each of the career roles. Alpha’s from the different scales were α = 0.75 (Maker role), α = 0.76 (Expert role), α = 0.78 (Presenter role), α = 0.82 (Guide role), α = 0.83 (Director role) and α = 0.75 (Inspirer role).

#### Control Variables

As in Study 1, demographic variables (age, sex, education) as well as work related variables (organizational tenure and job zone) were included as control variables in the analyses (Becker, 2005). Using the classification as provided by O^*^net (2019), job zone scores were obtained by having an independent rater assessing all jobs of respondents on the extent to which they needed experience and job training for job performance, using education level and the job description given by the respondents (1 = *no to little preparation or education is needed* to 5 = *extensive preparation and education is needed*). To calculate interrater reliability a second rater independently assessed 100 of the 285 jobs. Cohen’s kappa (*k* = 0.86, *SD* = 0.044) was excellent.

## Results Study 2

### Preliminary Analyses

Supplementary Table A9 presents the correlations, means and standard deviations of all study variables. Note that similar to Study 1, correlations indicate that personality traits are associated with career roles preferences and enactment in the expected direction (also see Wille et al., 2012).

### Mediation Analyses

To investigate the proposed mediating role of career role preferences in the relation between personality traits and career roles, we again used the PROCESS macro (Hayes, 2013). In each analysis the experienced enactment of one of the six roles was added as the dependent variable, the preference for that role was added as a mediator variable and the five personality variables were added as predictor variables^4^. Furthermore, age, sex, education, organizational tenure and job zone were included as a covariate in the mediation model (see Supplementary Tables A3–A8, last columns). As in Study 1, the findings support our overarching model that specific personality traits relate to career role preferences, subsequently resulting in career role enactment (see Figure 1). Below, we again describe the results for each of the hypotheses.

### Maker Role

Preference for the Maker role was positively related to Maker role enactment (see Supplementary Table A3). In addition, we found a positive relation between Conscientiousness and preference for the Maker role, and between Conscientiousness and perceived Maker role enactment. Furthermore, in line with Hypothesis 1, we found that the indirect effect of Conscientiousness via preference for the Maker role on perceived enactment of the Maker role was significant (Effect = 0.06, *SE* = 0.02, CI = [0.02; 0.12]). Other personality traits did not predict Maker role enactment.

### Expert Role

First, we found a significant positive relationship between preference for the Expert role and Expert role enactment (see Supplementary Table A4). Moreover, we found a significant positive relationship between Conscientiousness and preference for the Expert role. Furthermore, in line with Hypothesis 2a, we found that the indirect effect of Conscientiousness via preference for the Expert role on enactment of the Expert role was significant (Effect = 0.12, *SE* = 0.04, CI = [0.04; 0.21]).

Second, we found a significant positive relationship between Openness to experience and preference for the Expert role, and between Openness to experience and Expert role enactment. Furthermore, in line with Hypothesis 2b, we found that the indirect effect of Openness to experience via preference for the Expert role on enactment of the Expert role was significant (Effect = 0.20, *SE* = 0.06, CI = [0.10; 0.33]).

Unexpectedly, we found a negative relationship between Extraversion and preference for the Expert role. Furthermore, we found that the indirect effect of Extraversion via preference for the Expert role on enactment of the Expert role was significant (Effect = −0.10, *SE* = 0.03, CI = [−0.16;−0.05]).

### Presenter Role

For the Presenter role, we found a significant positive relationship between preference for this role and Presenter role enactment, between Extraversion and preference for the Presenter role, and between Extraversion and enactment of the Presenter role (see Supplementary Table A5). Furthermore, in line with Hypothesis 3, we found that the indirect effect of Extraversion via preference for the Presenter role on enactment of the Presenter role was significant (Effect = 0.10, *SE* = 0.03, CI = [0.04; 0.17]). Other personality traits did not predict Presenter role enactment.

### Guide Role

For the Guide role, we found a significant positive relationship between preference for this role and Guide role enactment, and between Agreeableness and preference for the Guide role, and between Agreeableness and enactment of the Guide role (see Supplementary Table A6). Furthermore, in line with Hypothesis 4, we found that the indirect effect of Agreeableness via preference for the Guide role on perceived enactment of the Guide role was significant (Effect = 0.12, *SE* = 0.04, CI = [0.06; 0.21]).

Unexpectedly, we found a significant positive relationship between Extraversion and preference for the Guide role, and between Extraversion and Guide role enactment. Furthermore, we found that the indirect effect of Extraversion via preference for the Guide role on enactment of the Guide role was significant (Effect = 0.04, *SE* = 0.02, CI = [0.02; 0.08]).

### Director Role

For the Director role, we found a significant positive relationship between preference for this role and Director role enactment, between Extraversion and preference for the Director role, and between Extraversion and Director role enactment (see Supplementary Table A7). Furthermore, in line with Hypothesis 5, we found that the indirect effect via preference for the Director role on enactment of the Director role was significant (Effect = 0.11, *SE* = 0.03, CI = [0.05; 0.18]). Other personality traits did not predict Director role enactment.

### Inspirer Role

First, we found a significant positive relationship between preference for the Inspirer role and Inspirer role enactment (see Supplementary Table A8). Moreover we found a significant positive relationship between Extraversion and preference for the Inspirer role, and between Extraversion and perceived Inspirer role enactment. Furthermore, in line with Hypothesis 6a, we found that the indirect effect via preference for the Inspirer role on enactment of the Inspirer role was significant (Effect = 0.07, *SE* = 0.02, CI = [0.03; 0.12]).

Second, we found a significant relationship between Openness to experience and preference for the Inspirer role, and between Openness to experience and Inspirer role enactment. Furthermore, in line with Hypothesis 6b, we found that the indirect effect via preference for the Inspirer role was significant (Effect = 0.11, *SE* = 0.04, CI = [0.04; 0.21]).

## Discussion

At work, employees have become increasingly responsible for shaping their careers (Savickas, 2013). The results of the present studies extend the knowledge of the relationship between individual personality traits and career role enactment by focusing on the mediating role of career role preferences. Results for the specific career roles are mostly in line with previous research findings and our hypotheses (Wille et al., 2012). The specific findings will be elaborated on below.

### Career Roles, Preferences, and Personality

In line with our expectations, we found the following effects in both studies. First, Conscientiousness was found to be the related to a preference for the Maker role, which, in turn predicted Maker role enactment (*Hypothesis 1*). This suggests that the possibility to pursue goals, an activity that aligns with the Conscientiousness domain, makes the Maker role more attractive (Denissen and Penke, 2008) because in the Maker role there is a strong focus on goals and mastery. Interestingly, although Wille et al. (2012) also expected Conscientiousness and engagement in the Maker role to be related, they did not find this relationship in their study. Second, Openness to experience (*Hypothesis 2b*) was a positive predictor of preference for and subsequent enactment of the Expert role. Apparently, the eagerness to explore, a sense of curiosity and the ability to think outside the box are important determinants of the willingness to and likelihood that someone will take on an Expert role. Third, Extraversion (*Hypothesis 3*) predicted enactment of the Presenter role via a preference for the Presenter role. This suggests that the ability and willingness to interact and connect with others, enhances peoples’ attraction to the Presenter role, because it allows them to engage in such activities. As a consequence, they are more likely to eventually end up in the Presenter role. Fourth, Agreeableness/Friendliness (*Hypothesis 4*) was positively related to preference for the Guide role, which, in turn, predicted Guide role enactment. This indicates that agreeable individuals will prefer and have a higher likelihood to end up in the Guide role, arguably because this allows them to interact with others and develop meaningful relationships (Judge et al., 2002). Fifth, Extraversion (*Hypothesis 5*) was positively related to Director role enactment, via a preference for this role. Apparently, characteristics such as expressiveness and assertiveness make roles that combine influencing others and gaining status particularly attractive (DeNeve and Cooper, 1998). Last, both Extraversion (*Hypothesis 6a*) and Openness to experience (*Hypothesis 6b*) were positively related to preference for and engagement in the Inspirer role. The Inspirer role may thus not only attract people who like to influence others, but also those who like to develop visions from a position of wonder, curiosity and exploration (Benoliel and Somech, 2014). We also expected Conscientiousness to be a positive predictor of preference for and subsequent enactment of the Expert role (*Hypothesis 2a*). However, we only find support for this hypothesis in Study 2. As this result only emerged in one study, caution with interpreting this result is warranted.

There were also some unexpected findings that emerged from our studies. First, results in both studies show that extraversion was positively related to a preference for, and subsequently enactment of the Guide role. Extraversion has been argued to be important for establishing interpersonal connections with others (Wiggins and Broughton, 1985; Wille et al., 2012). Because the Guide role entails forming connections and relationships with others it seems that, extraverted people – who are eager to connect with others-, prefer and end up in the Guide role. Second, some findings emerge from only one of the studies. Results from Study 1 show that Neuroticism was positively related to preference for the Guide role, which in turn predicted enactment of the Guide role. This finding is interesting, given the fact that previous research did not find strong links between Neuroticism and helping behavior (Barrick et al., 1992) or between Neuroticism and prosocial behavior (Habashi et al., 2016). However, recently, Guo et al. (2018) argued that in situations in which helping others requires less social skills, or when the social interaction is less anxiety-provoking, the negative association between Neuroticism and helping behavior may disappear. Instead, in these situations, other people’s suffering may also elicit more compassion and concern for others’ distress, which may promote neurotic individuals to behave prosocially. Perhaps this can explain why people high on Neuroticism ultimately ended up in the Guide Role in Study 1. Moreover, results from Study 2 show that Extraversion was negatively related to preference for, and subsequent enactment of the Expert role. Perhaps Extraverts find the Expert role less appealing because the Expert role requires little interpersonal contact and people in this role work mostly autonomous (Hurtz and Donovan, 2000; Hoekstra, 2011). Notably, although overall the two studies show very similar findings, these last results only appeared in one of the studies. One reason for these differential findings may be that the studies differed in the sample that was used. For Study 1, US workers were surveyed, whereas for Study 2 Dutch workers were surveyed. Although the used personality questionnaires were constructed to assess the personality of English and Dutch speaking respondents, respectively, research has shown that trait answering patterns can differ depending on culture or geographic location (Allik and McCrae, 2004: Melchers et al., 2016). In addition, although the convergent validity of both scales has been assessed (John et al., 2008; Hiemstra et al., 2011), the two scales may yield some differential relationships with outcome variables because of their differences. It remains unclear how the dissimilarities between the results of Study 1 and Study 2 should be interpreted as they could be the result from trait differences, response style differences or both (Melchers et al., 2016). Therefore, caution with interpreting these results is warranted.

Taken together, although there are some unexpected results, the overall pattern of our findings indicates that individual personality traits and resulting personal role preferences indeed play an important part in career role enactment. First, our results seem to show that especially Extraversion, Conscientiousness, and Openness to experience influence a broader range of role preferences, and subsequently career role enactment. Other traits, like Neuroticism and Agreeableness are less strongly related to role preferences and subsequent role enactment. This is in line with previous research showing the relative importance of specific personality traits over others (Wille et al., 2012). More importantly, our findings highlight the moderating mechanism through which personality traits can influence career role enactment. These findings testify to the importance of motivational processes for employee work behaviors (see Barrick et al., 2002). Indeed, peoples’ interest in certain roles is a good predictor of their actual enactment of these roles. As such, our findings underscore the importance of personal aspirations in how people shape their career. To understand career role enactment and long-term development, subsequent research may thus benefit, besides a focus on personality characteristics, from a focus on specific motivational processes such as personal preferences, goals and motives at work.

### Strengths, Limitations, and Future Research

The present research has both strengths and limitations. One strength is that our studies include both a US and a Dutch sample. That we find similar patterns of results points to generalizability of the study findings. Of course, we should be cautious generalizing the results to other populations (Bello et al., 2009), and more research with different populations would be welcome.

A limitation of our research is that Study 2 employed a cross-sectional design. Research has shown that the use of cross-sectional approaches to establish mediation effects can distort results (Maxwell and Cole, 2007). Specifically, such designs are often criticized for the risk of a common method bias and inability to infer causal relations (Podsakoff et al., 2003). Nonetheless, we opted for a cross-sectional design because we wanted to focus on peoples’ present knowledge of the self. That is, we were specifically interested in peoples *own* perceptions of their personality, preferences, and current role enactment. An alternative, as previously used by Wille et al. (2012), would have been retrospective research. However, retrospectively reporting on a career path (e.g., preferences and roles in the past) may be quite difficult and prone to bias (Miller et al., 1997). Moreover, for Study 1 we employed a 2-Wave research design and this study yielded similar results, which strengthens confidence in our findings. Nonetheless, future research could consider a number of options.

First, if time is not an issue, future research may consider testing the mediation hypotheses using a 3-Wave longitudinal design. Separating role preferences from the career role enactment measure and choosing longer time intervals would be beneficial as this provides opportunities to understand long-term effects of personality traits and role preferences for work outcomes. Moreover, longitudinal studies have been agued to allow for causal relationship analysis in complex designs (MacKinnon et al., 2002). Second, future studies could consider obtaining measures from different sources (e.g., collecting data from both employees and their supervisors, Van der Heijden et al., 2015) in order to reduce common method bias. This may be especially valuable because it has been argued that under the influence of self-enhancement or self-protection tactics people sometimes portray their actual role enactment less accurately (De Jong et al., 2014). Moreover, career roles are defined as “a coherent and enduring set of characteristics of the *perceived* effects of the way a person is doing his or her work” (Hoekstra, 2011, p. 165). This implies that it is not only self-perception that is important; others’ perceptions can be important as well. Notably, self-presentation tactics may also affect peoples’ reported career role preferences. For assessing career role preferences, future research may therefore also explore the usefulness of including measures of other peoples’ perceptions or use implicit measures of career role preferences as these may be less susceptible to social desirability (see Gadassi and Gati, 2009).

Notably, career development can be understood in terms of dynamic reciprocity (Rounds and Tracey, 1990), where environments are influenced by individuals and individuals are influenced by environments (Wood and Roberts, 2006; Wille et al., 2012). Specifically, career development can be seen as a gradual, interactive process that is the result of two simultaneous forces: *role pressure and granting* on the one hand, and *role taking* on the other (Hoekstra, 2011; Wille et al., 2012; De Jong et al., 2014). The current study focused solely on *role taking* processes (e.g., selecting fitting roles based on personal preferences), whereas career role enactment is also influenced by *role pressure* processes (Wille et al., 2012; De Jong et al., 2014). That is, external demands and expectations and environmental influences play a role in which career roles a person is expected to enact (Hoekstra, 2011). For example, employees may feel pressured into certain roles due to expectations from others about which roles people should take based on perceived personality characteristics. Notably, people’s perception of certain personality traits in others, are often biased and not necessarily correct (Zimmermann et al., 2018). We therefore would welcome longitudinal studies that investigate both *role taking* and *role pressure* processes, because this could help gain a better understanding of how employees integrate their own preferences with external pressures, by selecting, innovating and (re-) negotiating their roles (Parker, 2007).

In terms of career role enactment, and in line with the trait activation theory, it seems to be that *because* we feel good about expressing certain (preferred) traits that the role taking process is activated (Christiansen and Tett, 2008). Employees that are situated in a working environment that allows for the expression of individual interest and motives are thus rewarded for their behavior (Tett et al., 2013; Judge and Zapata, 2015). As such, a positive feedback loop may be created that will sustain the behavior through which employees end up in certain roles. However, although we have studied personality traits and career role preferences in career role enactment, we did not include the role of affect in our study. Future research could focus on the role of affective forecasting or mood in career role development in order to investigate if people indeed expect to feel better or actually feel better when they have the opportunity to enact the roles that are fitting to their personality.

Last, as mentioned previously it may be worthwhile to investigate differences between cultures (Cox et al., 1991; Melchers et al., 2016). For example, cultural background has been shown to affect peoples’ self-construal (Pekerti and Kwantes, 2011). In individualistic cultures (most Western countries), the emphasis lies on personal welfare and personal goals. In comparison, in collectivistic cultures (mostly non-Western and Asian countries) the focus is more on collective well-being and group goals (Markus and Kitayama, 1991). In both our studies, we used samples coming from individualistic cultures. Consequently, respondents in our studies may have been more inclined to behave according to their personal preferences and goals than respondents coming from more collectivistic cultures would have been. That is, it may be that the role of personality in career role enactment is greater in individualistic than in collectivistic countries. In collectivistic cultures, individuals are more likely to make an effort to fit into society and to pay heed to collective needs (Triandis, 1995). In such cultures, the influence of personality and personal preferences on the enactment of career roles may be relatively small. Up to date, these cultural differences have not yet been included in career role research. Thus, future research could include differences between cultures in career role enactment in order to gain more insight in career role theory.

### Practical Implications

As the current research suggests, personality traits and individual role preferences may influence the way that people behave in their work environment. Our findings have some tentative implications for organizations, HR professionals and employees. For example, HR-practices that acknowledge the fact that a certain job can be performed in multiple ways – in which different workers take on different roles- may benefit employees. In addition, the current study showed that peoples’ preferences are a good predictor of career role enactment. Organizations may consider tapping employees’ preferences in order to support role acquisition processes. Supporting environments that enable employees to perform work according to their preferences may lead to fulfilling career role enactment and prevent people from winding up in jobs that they do not like because they have to do the job in a certain way (Roberts and Caspi, 2003; Roberts, 2006). Practices that strengthen employees’ perception that there is a fit between themselves and the environment (for instance because that environment is supportive of employees’ personal preferences and strengths) may play an important role in increasing commitment and diminishing burnout or turnover amongst employees (Kristof-Brown et al., 2005; Pee and Min, 2017). Also important to note is that for most organizations to function effectively, it is not required that all employees can fulfill all roles. Likewise, there is not necessarily the need for all career roles to be equally distributed among the various jobs within the organization. This may provide employees some leeway in deciding on the career roles they want to take on. If given the chance, employees themselves are often motivated to select, optimize and develop their jobs and careers over time (Roberts and DelVecchio, 2000). Yet, not all employees may know how exactly they can do so. Organizations may consider to support their employees through counseling, coaching and job-crafting training in order to provide employees with the skills and strategies needed for change and personal development (Wrzesniewski and Dutton, 2001).

## Conclusion

There has been a growing recognition that individual characteristics shape and influence behaviors at work. In this article, we investigated the role of personality traits and preferences in career role enactment at work. By introducing the importance of personal preferences as a mediating mechanism between personality and career role enactment, we hope to contribute to a better understanding of how people come to occupy certain career roles. Having a clear understanding of the self, and ones preferences at work may help employees to select those roles that are congruent with their interests.

## Data Availability

The raw data supporting the conclusions of this manuscript will be made available by the authors, without undue reservation, to any qualified researcher.

## Ethics Statement

This research was carried out in accordance with the recommendations of the Heymans Institute Ethical Guidelines. The protocol was approved by the Ethical Committee of Psychology, University of Groningen. Online informed consent was obtained from all participants.

## Author Contributions

NdJ, BW, and KvdZ designed the study. NdJ collected and analyzed the data. NdJ and BW drafted the manuscript with NdJ taking the lead. JH and KvdZ engaged in several rounds of the critical feedback.

## Conflict of Interest Statement

The authors declare that the research was conducted in the absence of any commercial or financial relationships that could be construed as a potential conflict of interest.

## Footnotes

1 At Time 1, a total of 505 respondents completed the online questionnaire. We contacted these respondents again to participate at Time 2, and 302 respondents did so (of which five individuals did not participate at Time 1). From the initial sample of 297 workers that participated in both waves we deleted a number of respondents who indicated either not to use their data or who showed unusual response patterns (*N* = 18). We continued the analyses with the remaining 279 workers. To test for the selective dropout at Time 2, control variables (age, gender, education, employment, and job complexity) were used to compare continuers (*N* = 297) to dropouts (*N* = 208). However, the results showed no significant differences between both groups in terms of the control variables [gender: *F*(1,503) = 2.14, *p* = 0.14, age: *F*(1,503) = 1.21, *p* = 0.27, education: *F*(1,503) = 1.34, *p* = 0.25, employment: *F*(1,503) = 2.14, *p* = 0.27 and job complexity: *F*(1,503) = 0.037, *p* = 0.85].

2 Although all questionnaires have been validated in previous research we performed confirmatory factor analyses. Fit indices for the personality measurements were initially RMSEA = 0.092, CFI = 0.93 for Study 1 and RMSEA = 0.096, CFI = 0.83 for Study 2. Therefore, additional EFA analyses were conducted. Consequently, several items in the personality questionnaire (10 items for Study 2) were deleted, which resulted in an adequate fit structure for both personality measurements. We found adequate fit indices for career role enactment (RMSEA = 0.091, CFI = 0.96 for Study 1 and RMSEA = 0.082, CFI = 0.93 for Study 2) and for career role preferences (RMSEA = 0.10, CFI = 0.94 for Study 2). Notably, if we would have included all items for both personality questionnaires the conclusion of our analyses would have been the same as they are now.

3 The study scenarios are available by contacting the first author.

4 We conducted multiple mediation analysis to account for the multiple predictor variables. To add multiple predictor variables we used the seed = option in PROCESS. When using this option, mathematically all resulting paths, direct and indirect effects are as if they had all been estimated simultaneously (Hayes, 2013). Similar to previous research on personality characteristics and work outcomes we added all five personality traits at once (Akhtar et al., 2015). However, because we expected a specific career role preference to influence role enactment only the preference for the specific role was added as a mediator in the analyses. Note that we also conducted mediation analyses with a more complex model, including all different career role preferences as mediators. This did not change the pattern of the results, therefore we continued our analyses using the least complicated model.
