# Supplementary material for: Personality Traits and Career Role Enactment: Career Role Preferences as a Mediator
Source: Front Psychol. 2019 Jul 25;10:1720. doi: 10.3389/fpsyg.2019.01720 (PMC6671867; doi:10.3389/fpsyg.2019.01720)
Supplement: Supplementary file 1 [file Table_1.docx]

Table A1

*The Career Role Model: Six Career Roles.*

|  | Organizational performance domains | |
| --- | --- | --- |
|  | ExploitationProduction, results | ExplorationInnovation, change |
| **Dominant Personal motives** |  | |
| Distinction motivesAutonomy / Agency *Self – assertion* | **Maker** | **Expert** |
| *Integration motives*  *Connectedness, Belonging,*  *Cooperation, Sharing* | **Presenter** | **Guide** |
| ***Structure motives***  *Collective meaning, Cohesion,*  *Institutional Structure* | **Director** | **Inspirer** |

*Note.* From “A career roles model of career development” by H. A. Hoekstra, 2011, *Journal of Vocational Behavior, 78,* (2), 159-735. Copyright 2011 by Elsevier. Adapted with permission.
