# Supplementary material for: Personality Traits and Career Role Enactment: Career Role Preferences as a Mediator
Source: Front Psychol. 2019 Jul 25;10:1720. doi: 10.3389/fpsyg.2019.01720 (PMC6671867; doi:10.3389/fpsyg.2019.01720)
Supplement: Supplementary file 2 [file Table_2.docx]

Table A2

*Study 1 Descriptives and Correlations of Variables.*

| Variable | 1 | 2 | 3 | 4 | 5 | 6 | 7 | 8 | 9 | 10 | 11 | 12 | 13 | 14 | 15 | 16 | 17 | 18 | 19 | 20 | 21 | 22 |
| --- | --- | --- | --- | --- | --- | --- | --- | --- | --- | --- | --- | --- | --- | --- | --- | --- | --- | --- | --- | --- | --- | --- |
| 1 Age | 1 |  |  |  |  |  |  |  |  |  |  |  |  |  |  |  |  |  |  |  |  |  |
| 2 Sex*^a^* | .13^*^ | 1 |  |  |  |  |  |  |  |  |  |  |  |  |  |  |  |  |  |  |  |  |
| 3 Education*^b^* | -.02 | -.00 | 1 |  |  |  |  |  |  |  |  |  |  |  |  |  |  |  |  |  |  |  |
| 4 Job zone*^c^* | .12^*^ | -.07 | .54^**^ | 1 |  |  |  |  |  |  |  |  |  |  |  |  |  |  |  |  |  |  |
| 5 Employment | .88^**^ | .07 | -.14^*^ | .04 | 1 |  |  |  |  |  |  |  |  |  |  |  |  |  |  |  |  |  |
| *Career Roles* |  |  |  |  |  |  |  |  |  |  |  |  |  |  |  |  |  |  |  |  |  |  |
| 6 Enactment Maker | .23^**^ | -.00 | .08 | .21^**^ | .19^**^ | 1 |  |  |  |  |  |  |  |  |  |  |  |  |  |  |  |  |
| 7 Expert | .11 | -.12^*^ | .25^**^ | .38^**^ | .07 | .63^**^ | 1 |  |  |  |  |  |  |  |  |  |  |  |  |  |  |  |
| 8 Presenter | .04 | -.07 | .17^**^ | .23^**^ | .04 | .55^**^ | .71^**^ | 1 |  |  |  |  |  |  |  |  |  |  |  |  |  |  |
| 9 Guide | .01 | .10 | .07 | .02 | -.01 | .41^**^ | .39^**^ | .61^**^ | 1 |  |  |  |  |  |  |  |  |  |  |  |  |  |
| 10 Director | .05 | -.12^*^ | .21^**^ | .27^**^ | .04 | .57^**^ | .76^**^ | .82^**^ | .54^**^ | 1 |  |  |  |  |  |  |  |  |  |  |  |  |
| 11 Inspirer | .07 | -.07 | .12^*^ | .19^**^ | .05 | .54^**^ | .66^**^ | .79^**^ | .74^**^ | .77^**^ | 1 |  |  |  |  |  |  |  |  |  |  |  |
| 12 Preferences Maker | .10 | .12^*^ | .05 | .01 | .12^*^ | .33^**^ | .20^**^ | .23^**^ | .22^**^ | .20^**^ | .21^**^ | 1 |  |  |  |  |  |  |  |  |  |  |
| 13 Expert | -.03 | -.13^*^ | .19^**^ | .25^**^ | -.03 | .38^**^ | .63^**^ | .47^**^ | .27^**^ | .51^**^ | .45^**^ | .21^**^ | 1 |  |  |  |  |  |  |  |  |  |
| 14 Presenter | .00 | -.07 | .17^**^ | .08 | -.01 | .25^**^ | .41^**^ | .56^**^ | .37^**^ | .53^**^ | .45^**^ | .21^**^ | .33^**^ | 1 |  |  |  |  |  |  |  |  |
| 15 Guide | -.01 | .15^*^ | -.04 | -.12^*^ | .04 | .15^*^ | .10 | .28^**^ | .60^**^ | .27^**^ | .43^**^ | .22^**^ | .16^**^ | .31^**^ | 1 |  |  |  |  |  |  |  |
| 16 Director | -.02 | -.13^*^ | .14^*^ | .16^**^ | -.02 | .33^**^ | .47^**^ | .50^**^ | .28^**^ | .60^**^ | .46^**^ | .18^**^ | .47^**^ | .45^**^ | .17^**^ | 1 |  |  |  |  |  |  |
| 17 Inspirer | -.10 | -.02 | .02 | -.07 | -.11 | .17^*^ | .21^**^ | .44^**^ | .40^**^ | .39^**^ | .49^**^ | .12^*^ | .24^**^ | .41^**^ | .35^**^ | .33^**^ | 1 |  |  |  |  |  |
| *Individual Differences* |  |  |  |  |  |  |  |  |  |  |  |  |  |  |  |  |  |  |  |  |  |  |
| 18 Extraversion | .09 | -.08 | .11 | .19^**^ | .07 | .32^**^ | .39^**^ | .50^**^ | .32^**^ | .48^**^ | .47^**^ | .05 | .24^**^ | .28^**^ | .21^**^ | .28^**^ | .35^**^ | 1 |  |  |  |  |
| 19 Agreeableness | .12 | .02 | -.01 | -.03 | .11 | .30^**^ | .16^**^ | .24^**^ | .36^**^ | .20^*^ | .27^**^ | .13^*^ | .14^*^ | .11 | .37^**^ | .09 | .25^**^ | .31^**^ | 1 |  |  |  |
| 20 Conscientiousness | .25^**^ | .01 | -.08 | .12^*^ | .25^**^ | .43^**^ | .20^**^ | .19^*^ | .18^**^ | .20^**^ | .19^**^ | .30^**^ | .22^**^ | .13^*^ | .07 | .21^**^ | .09 | .23^**^ | .47^**^ | 1 |  |  |
| 21 Neuroticism | -.21 | .26^**^ | -.06 | -.16^**^ | -.22^**^ | -.33^*^ | -.35^**^ | -.34^**^ | -.16^**^ | -.34^**^ | -.31^**^ | -.09 | -.28^**^ | -.25^**^ | -.05 | -.29^**^ | -.18^**^ | -.45^**^ | -.53^**^ | -.53^**^ | 1 |  |
| 22 Openness to experience | .01 | -.15^*^ | .03 | .12^*^ | .01 | .39^**^ | .44^**^ | .40^**^ | .28^**^ | .39^**^ | .40^**^ | .07 | .37*^*^ | .25^**^ | .16^**^ | .25^**^ | .34^**^ | .51^**^ | .26^**^ | .26^**^ | -.38^**^ | 1 |
| Mean | 39.11 | 1.50 | 3.56 | 2.96 | 18.38 | 5.42 | 5.05 | 4.64 | 4.80 | 4.59 | 4.63 | 5.45 | 4.85 | 4.07 | 5.01 | 4.46 | 4.61 | 2.99 | 3.79 | 4.14. | 2.50 | 3.58 |
| SD | 10.80 | .51 | .98 | 1.17 | 11.23 | .91 | 1.16 | 1.26 | 1.28 | 1.35 | 1.20 | 1.18 | 1.58 | 1.59 | 1.48 | 1.62 | 1.64 | .94 | .72 | .66 | .97 | .71 |

*Note. N* = 279. *^a^* Sex (1= men, 2= women), *^b^*education (1= preliminary school, 2= high school, 3 = intermediate vocational education, 4 = higher vocational education, 5= university degree), *^c^*job zone (1 = little or no preparation needed, 2 = some preparation needed, 3 = medium preparation needed, 4 = considerable preparation needed, 5 = extensive preparation needed). *p<.05. (two-tailed), **p<.01. (two-tailed).
