# Supplementary material for: Personality Traits and Career Role Enactment: Career Role Preferences as a Mediator
Source: Front Psychol. 2019 Jul 25;10:1720. doi: 10.3389/fpsyg.2019.01720 (PMC6671867; doi:10.3389/fpsyg.2019.01720)
Supplement: Supplementary file 3 [file Table_3.docx]

Table A3

*Regression Results for the Indirect Effects of Study 1 and Study 2 with career role enactment of the Maker role as the dependent variable.*

|  | Mediator variable model (DV = Preference Maker role) | | | | | | | | | | | | | | |
| --- | --- | --- | --- | --- | --- | --- | --- | --- | --- | --- | --- | --- | --- | --- | --- |
| Predictor | Study 1*^a^* | | | | | | | Study 2*^b^* | | | | | | | |
|  | *b*^c^ | | SE | | *t* | | | *b*^c^ | | | SE | | | *t* | |
| Constant  Age  Sex  Education  Job zone  Employment  Neuroticism/ Stability*^d^*  Conscientiousness  Agreeableness/ Friendliness*^e^*  Extraversion  Openness to experience | 1.96  -.02  .22  .18  -.08  .02  .11  .62  .00  -.01  .07 | | .95  .01  .15  .09  .07  .01  .10  .13  .12  .09  .12 | | 2.06*  -1.30  1.48  2.06*  -1.08  1.71  1.05  4.68**  .01  -.13  .57 | | | 3.75  .00  .16  -.04  -.13  -.01  .00  .02  .00  -.00  .00 | | | .90  .01  .15  .09  .14  .01  .00  .01  .01  .00  .01 | | | 4.16**  .53  1.10  -.49  -99  -.72  .20  4.45**  .43  -.95  .34 | |
|  | Dependent variable model (DV = enactment of the Maker role) | | | | | | | | | | | | | | |
| Predictor | Study 1 | | | | | | | Study 2 | | | | | | | |
|  | *b^c^* | | SE | | | *t* | | *b^c^* | | | | SE | | *t* | |
| Constant  Age  Sex  Education  Job zone  Employment  Preference Maker role  Neuroticism/ Stability  Conscientiousness  Agreeableness/ Friendliness  Extraversion  Openness to experience | .68  .02  -.01  .01  .09  -.01  .17  .02  .30  .09  .08  .32 | | .63  .01  .10  .06  .05  .01  .04  .07  .09  .08  .06  .08 | | | 1.06  1.71  -.13  .12  1.87  -.67  4.20**  .28  3.30**  1.18  1.37  4.23** | | 11.39  -.12  3.23  -.59  -1.56  -.12  3.99  8.82  2.61  -.28  2.34  2.24 | | | | 9.79  .09  1.56  .94  1.43  .13  .00  .00  .01  .78  .02  .03 | | 1.16  -1.27  2.07*  -.62  -1.09  -.95  3.99**  8.82**  2.61**  -.28  2.34*  2.24* | |
|  | Indirect effects for preference in the Maker role for different personality characteristics | | | | | | | | | | | | | | |
|  | Study 1 | | | | | | | | Study 2 | | | | | | |
|  | Effect | Boot SE | | BootLLCI | | | BootULCI | | Effect | Boot SE | | | BootLLCI | | BootULCI |
| Neuroticism/ Stability | .02 | .02 | | -.01 | | | .06 | | .00 | .01 | | | -.02 | | .03 |
| Conscientiousness | .11 | .04 | | .04 | | | .20 | | .06 | .02 | | | .02 | | .12 |
| Agreeableness/ Friendliness | .00 | .02 | | -.04 | | | .05 | | .01 | .02 | | | -.03 | | .04 |
| Extraversion | -.00 | .02 | | -.03 | | | .03 | | -.01 | .01 | | | -.04 | | .01 |
| Openness to experience | .01 | .02 | | -.03 | | | .06 | | .01 | .02 | | | -.03 | | .04 |

*Note.* Bootstrap (Boot) sample size = 10.000. Level of confidence interval = 95%. *^a^N_study 1_* = 279*, ^b^N_study 2_* = 285. *^c^*Unstandardized regression coefficients. *^d,e^*Variables differ in the mediation model presented in Study 1 compared to Study 2, both are shown in the table.^*^ *p* < .05. ^**^ *p* < .01.
