# Supplementary material for: Personality Traits and Career Role Enactment: Career Role Preferences as a Mediator
Source: Front Psychol. 2019 Jul 25;10:1720. doi: 10.3389/fpsyg.2019.01720 (PMC6671867; doi:10.3389/fpsyg.2019.01720)
Supplement: Supplementary file 4 [file Table_4.docx]

Table A4

*Regression Results for the Indirect Effects of Study 1 and Study 2 with career role enactment of the Expert role as the dependent variable.*

|  | Mediator variable model (DV = Preference Expert role) | | | | | | | | | | | | | | |
| --- | --- | --- | --- | --- | --- | --- | --- | --- | --- | --- | --- | --- | --- | --- | --- |
| Predictor | Study 1*ª* | | | | | | | Study 2*^b^* | | | | | | | |
|  | *b^c^* | | SE | | *t* | | | *b^c^* | | | SE | | | *t* | |
| Constant  Age  Sex  Education  Job zone  Employment  Neuroticism/ Stability*^d^*  Conscientiousness  Agreeableness/ Friendliness*^e^*  Extraversion  Openness to experience | 1.88  -.02  -.11  .20  .17  .01  -.19  .27  -.08  -.01  .63 | | 1.20  .02  .18  .11  .09  .02  .13  .17  .15  .11  .14 | | 1.56  -1.18  -.58  1.86  1.82  .47  -1.45  1.60  -.53  -.05  4.35** | | | 2.55  -.00  -.23  .12  .04  .00  -.01  .02  -.01  -.01  .03 | | | .84  .01  .14  .08  .13  .01  .00  .00  .01  .00  .01 | | | 3.04**  -.20  -1.63  -1.50  .32  .31  -1.34  3.66**  -0.91  -3.62**  4.92** | |
|  | Dependent variable model (DV = enactment of the Expert role) | | | | | | | | | | | | | | |
| Predictor | Study 1 | | | | | | | Study 2 | | | | | | | |
|  | *b^c^* | | SE | | | *t* | | *b^c^* | | | | SE | | *t* | |
| Constant  Age  Sex  Education  Job zone  Employment  Preference Expert role  Neuroticism/ Stability  Conscientiousness  Agreeableness/ Friendliness  Extraversion  Openness to experience | 1.54  .01  -.00  .04  .18  .00  .36  -.09  -.10  -.01  .15  .27 | | .69  .01  .11  .06  .05  .01  .03  .07  .10  .09  .06  .09 | | | 2.23*  .91  -.04  .65  3.49**  .00  10.21**  -1.25  -1.04  -.09  2.26*  3.20** | | -5.27  -.12  -2.44  -1.38  .55  -.04  6.62  -.08  .13  -.17  .09  .48 | | | | 12.00  .11  1.95  1.17  1.78  .16  .86  .06  .07  .08  .06  .09 | | -.44  -1.05  -1.26  1.18  .31  -.23  7.69**  -1.17  1.95  -2.01*  1.62  5.31** | |
|  | Indirect effects for preference in the Expert role for different personality characteristics | | | | | | | | | | | | | | |
|  | Study 1 | | | | | | | | Study 2 | | | | | | |
|  | Effect | Boot SE | | BootLLCI | | | BootULCI | | Effect | Boot SE | | | BootLLCI | | BootULCI |
| Neuroticism/ Stability | -.07 | .05 | | -.17 | | | .03 | | -.04 | .03 | | | -.10 | | .02 |
| Conscientiousness | .10 | .06 | | -.03 | | | .22 | | .12 | .04 | | | .04 | | .21 |
| Agreeableness/ Friendliness | -03 | .06 | | -.14 | | | .09 | | -.04 | .04 | | | -.12 | | .04 |
| Extraversion | -.00 | .04 | | -.09 | | | .08 | | -.10 | .03 | | | -.16 | | -.05 |
| Openness to experience | .23 | .06 | | .12 | | | .36 | | .20 | .06 | | | .10 | | .33 |

*Note.* Bootstrap (Boot) sample size = 10.000. Level of confidence interval = 95%. *^a^N_study 1_* = 279*, ^b^N_study 2_* = 285. *^c^*Unstandardized regression coefficients. *^d,e^*Variables differ in the mediation model presented in Study 1 compared to Study 2, both are shown in the table.^*^ *p* < .05. ^**^ *p* < .01.
