# Supplementary material for: Personality Traits and Career Role Enactment: Career Role Preferences as a Mediator
Source: Front Psychol. 2019 Jul 25;10:1720. doi: 10.3389/fpsyg.2019.01720 (PMC6671867; doi:10.3389/fpsyg.2019.01720)
Supplement: Supplementary file 5 [file Table_5.docx]

Table A5

*Regression Results for the Indirect Effects of Study 1 and Study 2 with career role enactment of the Presenter role as the dependent variable.*

|  | Mediator variable model (DV = Preference Presenter role) | | | | | | | | | | | | | | |
| --- | --- | --- | --- | --- | --- | --- | --- | --- | --- | --- | --- | --- | --- | --- | --- |
| Predictor | Study 1*ª* | | | | | | | Study 2*^b^* | | | | | | | |
|  | *b^c^* | | SE | | *t* | | | *b^c^* | | | SE | | | *t* | |
| Constant  Age  Sex  Education  Job zone  Employment  Neuroticism/ Stability*^d^*  Conscientiousness  Agreeableness/ Friendliness*^e^*  Extraversion  Openness to experience | 2.62  -.01  .01  .32  -.14  .00  -.25  .10  -.17  .28  .28 | | 1.26  .02  .19  .11  .10  .02  .14  .18  .16  .12  .15 | | 2.07*  -.49  .07  2.79**  -1.42  .18  -1.85  .58  -1.05  2.36*  1.83 | | | 1.21  .01  -.02  .10  -.04  -.01  .01  .01  -.00  .03  .01 | | | .73  .01  .12  .07  .11  .01  .00  .00  .01  .00  .01 | | | 1.65  1.04  .15  1.38  -.40  -1.03  -.73  1.73  -.19  7.71**  1.20 | |
|  | Dependent variable model (DV = enactment of the Presenter role) | | | | | | | | | | | | | | |
| Predictor | Study 1 | | | | | | | Study 2 | | | | | | | |
|  | *b^c^* | | SE | | | *t* | | *b^c^* | | | | SE | | *t* | |
| Constant  Age  Sex  Education  Job zone  Employment  Preference Presenter role  Neuroticism/ Stability  Conscientiousness  Agreeableness/ Friendliness  Extraversion  Openness to experience | .97  -.01  .04  -.02  .17  .01  .35  -.03  -.08  .16  .34  .22 | | .79  .01  .12  .07  .06  .01  .04  .09  .11  .10  .08  .09 | | | 1.23  -1.08  .32  -.29  2.85**  1.01  9.14**  -.32  -.71  1.68  4.57**  2.37* | | -20.46  .13  -1.85  2.82  -.77  -.03  3.63  .10  .10  .08  .27  .25 | | | | 11.02  .11  1.80  1.09  1.65  .15  .92  .06  .06  .08  .06  .08 | | -1.86  1.25  -1.03  2.60**  -.46  -.19  3.95**  1.63  1.58  1.06  4.60**  3.19** | |
|  | Indirect effects for preference in the Presenter role for different personality characteristics | | | | | | | | | | | | | | |
|  | Study 1 | | | | | | | | Study 2 | | | | | | |
|  | Effect | Boot SE | | BootLLCI | | | BootULCI | | Effect | Boot SE | | | BootLLCI | | BootULCI |
| Neuroticism/ Stability | -.09 | .06 | | -.21 | | | .02 | | -.01 | .01 | | | -.04 | | .02 |
| Conscientiousness | .04 | .07 | | -.10 | | | .16 | | .03 | .02 | | | -.00 | | .07 |
| Agreeableness/ Friendliness | -.06 | .06 | | -.18 | | | .07 | | -.00 | .02 | | | -.05 | | .04 |
| Extraversion | .10 | .04 | | .02 | | | .18 | | .10 | .03 | | | .04 | | .17 |
| Openness to experience | .10 | .06 | | -.02 | | | .23 | | .02 | .02 | | | -.01 | | .08 |

*Note.* Bootstrap (Boot) sample size = 10.000. Level of confidence interval = 95%. *^a^N_study 1_* = 279*, ^b^N_study 2_* = 285. *^c^*Unstandardized regression coefficients. *^d,e^*Variables differ in the mediation model presented in Study 1 compared to Study 2, both are shown in the table.^*^ *p* < .05. ^**^ *p* < .01.
