# Supplementary material for: Personality Traits and Career Role Enactment: Career Role Preferences as a Mediator
Source: Front Psychol. 2019 Jul 25;10:1720. doi: 10.3389/fpsyg.2019.01720 (PMC6671867; doi:10.3389/fpsyg.2019.01720)
Supplement: Supplementary file 6 [file Table_6.docx]

Table A6

*Regression Results for the Indirect Effects of Study 1 and Study 2 with career role enactment of the Guide role as the dependent variable.*

|  | Mediator variable model (DV = Preference Guide role) | | | | | | | | | | | | | | |
| --- | --- | --- | --- | --- | --- | --- | --- | --- | --- | --- | --- | --- | --- | --- | --- |
| Predictor | Study 1*^a^* | | | | | | | Study 2*^b^* | | | | | | | |
|  | *b^c^* | | SE | | *t* | | | *b^c^* | | | SE | | | *t* | |
| Constant  Age  Sex  Education  Job zone  Employment  Neuroticism/ Stability*^d^*  Conscientiousness  Agreeableness/ Friendliness*^e^*  Extraversion  Openness to experience | .04  -.02  .34  .05  -.14  .02  .33  -.14  .88  .27  .18 | | 1.10  .02  .17  .10  .08  .02  .12  .15  .14  .10  .13 | | .04  -1.28  2.02*  .53  -1.65  .147  2.79**  -.89  6.42**  2.58*  1.38 | | | 1.40  .01  .09  .09  -.04  -.01  -.01  -.00  .04  .01  .00 | | | .79  .01  .13  .08  .12  .01  .00  .00  .01  .00  .01 | | | 1.79  1.44  .71  1.21  -.32  -.66  -1.92  -1.04  6.50**  3.48**  .66 | |
|  | Dependent variable model (DV = enactment of the Guide role) | | | | | | | | | | | | | | |
| Predictor | Study 1 | | | | | | | Study 2 | | | | | | | |
|  | *b^c^* | | SE | | | *t* | | *b^c^* | | | | SE | | *t* | |
| Constant  Age  Sex  Education  Job zone  Employment  Preference Guide role  Neuroticism/ Stability  Conscientiousness  Agreeableness/ Friendliness  Extraversion  Openness to experience | -.53  .01  .09  .08  .01  .02  .46  .04  .13  .17  .15  .19 | | .84  .01  .13  .08  .06  .01  .05  .09  .12  .11  .08  .10 | | | -.64  .98  .73  1.04  .22  -1.39  9.81**  .38  1.12  1.51  1.93*  1.90 | | -25.64  .04  2.65  1.77  -.53  .16  3.34  .10  .06  .32  .29  .27 | | | | 10.37  .10  1.69  1.02  1.55  .14  .80  .06  .06  .08  .05  .07 | | -2.47*  .43  1.57  1.73  -.34  1.16  4.16**  1.75  1.08  3.97**  5.78**  3.44** | |
|  | Indirect effects for preference in the Guide role for different personality characteristics | | | | | | | | | | | | | | |
|  | Study 1 | | | | | | | | Study 2 | | | | | | |
|  | Effect | Boot SE | | BootLLCI | | | BootULCI | | Effect | Boot SE | | | BootLLCI | | BootULCI |
| Neuroticism/ Stability | .15 | .06 | | .05 | | | .27 | | -.03 | .01 | | | -.06 | | -.00 |
| Conscientiousness | -.06 | .07 | | -.21 | | | .08 | | -.02 | .02 | | | -.06 | | .02 |
| Agreeableness/ Friendliness | .40 | .09 | | .25 | | | .59 | | .12 | .04 | | | .06 | | .21 |
| Extraversion | .12 | .05 | | .03 | | | .22 | | .04 | .02 | | | .02 | | .08 |
| Openness to experience | .08 | .07 | | -.06 | | | .23 | | .01 | .02 | | | -.03 | | .06 |

*Note.* Bootstrap (Boot) sample size = 10.000. Level of confidence interval = 95%. *^a^N_study 1_* = 279*, ^b^N_study 2_* = 285. *^c^*Unstandardized regression coefficients. *^d,e^*Variables differ in the mediation model presented in Study 1 compared to Study 2, both are shown in the table.^*^ *p* < .05. ^**^ *p* < .01.
