# Supplementary material for: Personality Traits and Career Role Enactment: Career Role Preferences as a Mediator
Source: Front Psychol. 2019 Jul 25;10:1720. doi: 10.3389/fpsyg.2019.01720 (PMC6671867; doi:10.3389/fpsyg.2019.01720)
Supplement: Supplementary file 7 [file Table_7.docx]

Table A7

*Regression Results for the Indirect Effects of Study 1 and Study 2 with career role enactment of the Director role as the dependent variable.*

|  | Mediator variable model (DV = Preference Director role) | | | | | | | | | | | | | | |
| --- | --- | --- | --- | --- | --- | --- | --- | --- | --- | --- | --- | --- | --- | --- | --- |
| Predictor | Study 1*ª* | | | | | | | Study 2*^b^* | | | | | | | |
|  | *b^c^* | | SE | | *t* | | | *b ^c^* | | | SE | | | *t* | |
| Constant  Age  Sex  Education  Job zone  Employment  Neuroticism/ Stability*^d^*  Conscientiousness  Agreeableness/ Friendliness*^e^*  Extraversion  Openness to experience | 3.06  -.01  -.15  .18  .03  -.00  -.27  .37  -.29  .27  .21 | | 1.27  .02  .20  .11  .10  .02  .14  .18  .16  .12  .15 | | 2.40*  -.53  -.78  1.59  .28  -.14  -1.97*  2.09*  -1.80  2.27*  1.38 | | | .06  .02  -.16  .12  .11  -.01  .01  .01  -.00  .02  .01 | | | .85  .01  .14  .08  .13  .01  .00  .00  .01  .00  .01 | | | .08  1.93  -1.16  1.46  .85  -1.13  2.02*  1.63  -.39  3.76**  2.18* | |
|  | Dependent variable model (DV = enactment of the Director role) | | | | | | | | | | | | | | |
| Predictor | Study 1 | | | | | | | Study 2 | | | | | | | |
|  | *b^c^* | | SE | | | *t* | | *b^c^* | | | | SE | | *t* | |
| Constant  Age  Sex  Education  Job zone  Employment  Preference Director role  Neuroticism/ Stability  Conscientiousness  Agreeableness/ Friendliness  Extraversion  Openness to experience | .25  -.01  -.05  .09  .12  .01  .39  -.00  -.09  .14  .34  .24 | | .84  .01  .13  .08  .06  .01  .04  .09  .12  .10  .08  .10 | | | .30  -.49  -.36  1.25  1.83  .89  9.88**  -.04  -.78  1.32  4.35**  2.37* | | -21.36  .18  -.77  2.63  -.02  -.29  7.05  .12  -.00  -.06  .22  .23 | | | | 12.86  .12  2.11  1.27  1.94  .18  .93  .07  .07  .09  .06  .09 | | -1.66  1.48  -.37  2.06  -.01  -1.62  7.61**  1.70  -.02  -.62  3.42*  2.43* | |
|  | Indirect effects for preference in the Director role for different personality characteristics | | | | | | | | | | | | | | |
|  | Study 1 | | | | | | | | Study 2 | | | | | | |
|  | Effect | Boot SE | | BootLLCI | | | BootULCI | | Effect | Boot SE | | | BootLLCI | | BootULCI |
| Neuroticism/ Stability | -11 | .07 | | -.24 | | | .02 | | .07 | .03 | | | .00 | | .13 |
| Conscientiousness | .15 | .08 | | -.01 | | | .31 | | .06 | .04 | | | -.02 | | .13 |
| Agreeableness/ Friendliness | -.11 | .07 | | -.26 | | | .03 | | -.02 | .04 | | | -.10 | | .07 |
| Extraversion | .11 | .05 | | .01 | | | .21 | | .11 | .03 | | | .05 | | .18 |
| Openness to experience | .08 | .08 | | -.06 | | | .24 | | .09 | .05 | | | .00 | | .20 |

*Note.* Bootstrap (Boot) sample size = 10.000. Level of confidence interval = 95%. *^a^N_study 1_* = 279*, ^b^N_study 2_* = 285. *^c^*Unstandardized regression coefficients. *^d,e^*Variables differ in the mediation model presented in Study 1 compared to Study 2, both are shown in the table.^*^ *p* < .05. ^**^ *p* < .01.
