# Supplementary material for: Personality Traits and Career Role Enactment: Career Role Preferences as a Mediator
Source: Front Psychol. 2019 Jul 25;10:1720. doi: 10.3389/fpsyg.2019.01720 (PMC6671867; doi:10.3389/fpsyg.2019.01720)
Supplement: Supplementary file 8 [file Table_8.docx]

Table A8

*Regression Results for the Indirect Effects of Study 1 and Study 2 with career role enactment of the Inspirer role as the dependent variable.*

|  | Mediator variable model (DV = Preference Inspirer role) | | | | | | | | | | | | | | |
| --- | --- | --- | --- | --- | --- | --- | --- | --- | --- | --- | --- | --- | --- | --- | --- |
| Predictor | Study 1*^a^* | | | | | | | Study 2*^b^* | | | | | | | |
|  | *b^c^* | | SE | | *t* | | | *b^c^* | | | SE | | | *t* | |
| Constant  Age  Sex  Education  Job zone  Employment  Neuroticism/ Stability *^d^*  Conscientiousness  Agreeableness/Friendliness*^e^*  Extraversion  Openness to experience | 1.08  -.00  .11  .10  -.21  -.02  .01  -.07  .34  .43  .49 | | 1.25  .02  .19  .11  .09  .02  .14  .17  .16  .12  .15 | | .86  -.13  .59  .86  -2.23*  -.90  .10  -.39  2.15*  3.64**  3.23** | | | .40  .02  -.11  .14  -.03  -.02  -.00  -.00  .01  .02  .03 | | | .75  .01  .12  .07  .11  .01  .00  .00  .01  .01  .01 | | | .53  2.29*  -.89  1.85  -.30  -1.57*  -.57  -.66  1.03  4.83**  5.16** | |
|  | Dependent variable model (DV = enactment of the Inspirer role) | | | | | | | | | | | | | | |
| Predictor | Study 1 | | | | | | | Study 2 | | | | | | | |
|  | *b^c^* | | SE | | | *t* | | *b^c^* | | | | SE | | *t* | |
| Constant  Age  Sex  Education  Job zone  Employment  Preference Inspirer role  Neuroticism/ Stability  Conscientiousness  Agreeableness/Friendliness  Extraversion  Openness to experience | .86  .01  -.05  .02  .15  .00  .26  -.03  .01  .10  .27  .20 | | .81  .01  .12  .07  .06  .01  .04  .09  .11  .10  .08  .10 | | | 1.06  .45  -.37  .25  2.33*  .14  6.62**  -.36  .13  .96  3.42**  2.02* | | -5.37  .17  2.42  .18  -1.81  -.18  3.74  .06  .07  .15  .32  .16 | | | | 9.59  .09  1.57  .95  1.44  .13  .78  .05  .05  .07  .05  .07 | | -.56  1.78  1.54  .19  -1.26  -1.39  4.80**  1.19  1.27  2.20*  6.78**  2.24** | |
|  | Indirect effects for preference in the Inspirer role for different personality characteristics | | | | | | | | | | | | | | |
|  | Study 1 | | | | | | | | Study 2 | | | | | | |
|  | Effect | Boot SE | | BootLLCI | | | BootULCI | | Effect | Boot SE | | | BootLLCI | | BootULCI |
| Neuroticism/ Stability | .00 | .04 | | -.07 | | | .09 | | -.01 | .02 | | | -.04 | | .02 |
| Conscientiousness | -.02 | .05 | | -.12 | | | .09 | | -.01 | .02 | | | -.05 | | .02 |
| Agreeableness/Friendliness | .09 | .05 | | .00 | | | .20 | | .02 | .02 | | | -.02 | | .08 |
| Extraversion | .11 | .04 | | .05 | | | .20 | | .07 | .02 | | | .03 | | .12 |
| Openness to experience | .13 | .06 | | .03 | | | .26 | | .11 | .04 | | | .04 | | .21 |

*Note.* Bootstrap (Boot) sample size = 10.000. Level of confidence interval = 95%. *^a^N_study 1_* = 279*, ^b^N_study 2_* = 285. *^c^*Unstandardized regression coefficients. *^d,e^*Variables differ in the mediation model presented in Study 1 compared to Study 2, both are shown in the table.^*^ *p* < .05. ^**^ *p* < .01.
