# Supplementary material for: Personality Traits and Career Role Enactment: Career Role Preferences as a Mediator
Source: Front Psychol. 2019 Jul 25;10:1720. doi: 10.3389/fpsyg.2019.01720 (PMC6671867; doi:10.3389/fpsyg.2019.01720)
Supplement: Supplementary file 9 [file Table_9.docx]

Table A9

*Study 2 Descriptives and Correlations of Variables.*

| Variable | 1 | 2 | 3 | 4 | 5 | 6 | 7 | 8 | 9 | 10 | 11 | 12 | 13 | 14 | 15 | 16 | 17 | 18 | 19 | 20 | 21 | 22 |
| --- | --- | --- | --- | --- | --- | --- | --- | --- | --- | --- | --- | --- | --- | --- | --- | --- | --- | --- | --- | --- | --- | --- |
| 1 Age | 1 |  |  |  |  |  |  |  |  |  |  |  |  |  |  |  |  |  |  |  |  |  |
| 2 Sex*^a^* | -.08 | 1 |  |  |  |  |  |  |  |  |  |  |  |  |  |  |  |  |  |  |  |  |
| 3 Education*^b^* | -.19^*^ | .04 | 1 |  |  |  |  |  |  |  |  |  |  |  |  |  |  |  |  |  |  |  |
| 4 Job zone*^c^* | -.14^*^ | -.09 | .39^**^ | 1 |  |  |  |  |  |  |  |  |  |  |  |  |  |  |  |  |  |  |
| 5 Employment | .52^*^ | -.07 | -.28^**^ | -.13^*^ | 1 |  |  |  |  |  |  |  |  |  |  |  |  |  |  |  |  |  |
| *Career Roles* |  |  |  |  |  |  |  |  |  |  |  |  |  |  |  |  |  |  |  |  |  |  |
| 6 Enactment Maker | -.05 | .21^**^ | -.10 | -.17^**^ | -.09 | 1 |  |  |  |  |  |  |  |  |  |  |  |  |  |  |  |  |
| 7 Expert | -.05 | -.04 | .08 | .06 | -.09 | .28^**^ | 1 |  |  |  |  |  |  |  |  |  |  |  |  |  |  |  |
| 8 Presenter | -.05 | -.01 | .07 | -.01 | -.09 | .34^**^ | .23^**^ | 1 |  |  |  |  |  |  |  |  |  |  |  |  |  |  |
| 9 Guide | .06 | .14^*^ | .00 | -.03 | -.04 | .29^**^ | .06 | .58^**^ | 1 |  |  |  |  |  |  |  |  |  |  |  |  |  |
| 10 Director | .07 | -.05 | .12 | .07 | -.15^**^ | .28^**^ | .07 | .67^**^ | .58^**^ | 1 |  |  |  |  |  |  |  |  |  |  |  |  |
| 11 Inspirer | .09 | .12^*^ | -.06 | -.10 | -.13^*^ | .39^**^ | .12^*^ | .70^**^ | .68^**^ | .62^**^ | 1 |  |  |  |  |  |  |  |  |  |  |  |
| 12 Preferences Maker | .04 | .12^*^ | -.06 | -.13^*^ | .01 | .35^**^ | .07 | .07 | .03 | -.02 | .08 | 1 |  |  |  |  |  |  |  |  |  |  |
| 13 Expert | .02 | -.06 | .09 | .05 | .01 | .09 | .49^**^ | -.08 | -.10 | -.13^*^ | -.09 | .43^**^ | 1 |  |  |  |  |  |  |  |  |  |
| 14 Presenter | -.03 | .09 | .02 | -.03 | -.13^*^ | .21^**^ | .16^**^ | .43^**^ | .35^**^ | .36^**^ | .43^**^ | .38^**^ | .30^**^ | 1 |  |  |  |  |  |  |  |  |
| 15 Guide | .07 | .13^*^ | .01 | -.01 | -.06 | .11 | .07 | .16^**^ | .44^**^ | .12^*^ | .28^**^ | .37^**^ | .35^**^ | .58^**^ | 1 |  |  |  |  |  |  |  |
| 16 Director | .07 | -.08 | .07 | .07 | -.08 | .22^**^ | .03 | .39^**^ | .33^**^ | .53 | .38^**^ | .38^**^ | .29^**^ | .64^**^ | .45^**^ | 1 |  |  |  |  |  |  |
| 17 Inspirer | .08 | -.02 | .06 | .02 | -.13^*^ | .15^*^ | .17^**^ | .41^**^ | .39^**^ | .40 | .49^**^ | .38^**^ | .41^**^ | .79^**^ | .65^**^ | .71^**^ | 1 |  |  |  |  |  |
| *Individual Differences* |  |  |  |  |  |  |  |  |  |  |  |  |  |  |  |  |  |  |  |  |  |  |
| 18 Extraversion | -.13^*^ | .12^*^ | -.09 | -.04 | -.17^**^ | .22^**^ | .09 | .45^**^ | .47^**^ | .34^**^ | .53^**^ | -.02 | -.17^**^ | .48^**^ | .28^**^ | .28^**^ | .37^**^ | 1 |  |  |  |  |
| 19 Friendliness | .15^*^ | .09 | -.09 | -.04 | -.01 | .19^**^ | .04 | .24^**^ | .47^**^ | .12^*^ | .34^**^ | .09 | .01 | .15^*^ | .44^**^ | .11 | .23^**^ | .23^**^ | 1 |  |  |  |
| 20 Conscientiousness | -.01 | .21^**^ | -.07 | -.16^**^ | .02 | .54^**^ | .20^**^ | .14^*^ | .15^*^ | .04 | .14^*^ | .31^**^ | .23^**^ | .11 | .03 | .09 | .02 | .03 | .19^**^ | 1 |  |  |
| 21 Stability | .14^*^ | -.31^**^ | .00 | -.00 | -.00 | .13^*^ | .03 | .21^**^ | .16^**^ | .26^**^ | .17^**^ | -.00 | .00 | .04 | -.04 | .23^**^ | .13^*^ | .14^*^ | .09 | .01 | 1 |  |
| 22 Openness to experience | .09 | -.01 | -.05 | -.00 | -.09 | .28^**^ | .39^**^ | .38^**^ | .41^**^ | .32^**^ | .41^**^ | .06 | .24*^*^ | .23^**^ | .20^**^ | .27^**^ | .42^**^ | .31^**^ | .34^**^ | .19^**^ | .25^**^ | 1 |
| Mean | 41 | .46 | 4.02 | 3.82 | 7.68 | 72.84 | 64.91 | 63.38 | 71.73 | 59.74 | 65.43 | 5.08 | 4.95 | 4.19 | 4.96 | 4.43 | 4.69 | 64.85 | 70.81 | 72.19 | 60.24 | 77.72 |
| SD | 9.53 | .50 | .85 | .55 | 6.60 | 15.41 | 18.01 | 16.41 | 16.64 | 19.66 | 15.69 | 1.15 | 1.11 | 1.02 | 1.09 | 1.13 | 1.06 | 17.10 | 11.33 | 13.88 | 15.21 | 11.65 |

*Note. N* = 285, *^a^*Sex (0= men, 1= women), *^b^*education (1= preliminary school, 2= high school, 3 = intermediate vocational education, 4 = higher vocational education, 5= university degree), *^c^*job zone (1 = little or no preparation needed, 2 = some preparation needed, 3 = medium preparation needed, 4 = considerable preparation needed, 5 = extensive preparation needed). *p < .05 (two-tailed), **p < .01 (two-tailed).
